# Long-term integrated soil-crop management improves soil microbial community structure to reduce GHG emission and increase yield

**DOI:** 10.3389/fmicb.2022.1024686

**Published:** 2022-10-25

**Authors:** Ningning Yu, Jiai Liu, Baizhao Ren, Bin Zhao, Peng Liu, Zheng Gao, Jiwang Zhang

**Affiliations:** ^1^State Key Laboratory of Crop Biology and College of Agronomy, Shandong Agricultural University, Taian, Shandong, China; ^2^State Key Laboratory of Crop Biology and College of Life Sciences, Shandong Agricultural University, Taian, Shandong, China

**Keywords:** soil bacterial community, soil characteristics, greenhouse gas emissions, carbon and nitrogen cycle, yield, integrated soil-crop management

## Abstract

Integrated soil-crop management (ISCM) has been shown as an effective strategy to increase efficiency and yield while its soil microbial community structure and function remain unclear. We evaluated changes in soil physicochemical factors, bacterial community structure responses, and the contributions of soil properties and bacterial communities to summer maize-winter wheat yield and GHG emissions through an ISCM experiment [T1 (local smallholder farmers practice system), T2 (improved management system), T3 (high–yield production system), and T4 (optimized management system)], which could provide scientific guidance for sustainable development of soil in summer maize-winter wheat rotation system. The results showed that the optimized ISCM could improve the soil quality, which significantly changed the soil bacterial community structure to reduce GHG emissions and increase yield. The co-occurrence network density of T3 was increased significantly. The Acidobacteria (class) and OM190 (class) were enriched in T2 and T4. The Frankiales (order) and Gaiellales (order) were enriched in T3. However, the changes in different crop growth stages were different. At the wheat jointing stage and maize mature stage, T4 could enhance carbon-related functional groups, such as aromatic hydrocarbon degradation and hydrocarbon degradation, to increase the soil organic carbon content. And at the maize tasseling stage, T4 could enhance nitrogen-related functional groups. And soil bacteria structure and function indirectly affected annual yield and GHG emission. T2 and T4 exhibited a similar soil microbial community. However, the yield and nitrogen use efficiency of T2 were reduced compared to those of T4. The yield of T3 was the highest, but the GHG emission increased and soil pH and nitrogen use efficiency decreased significantly. Therefore, T4 was a suitable management system to improve soil quality and soil bacterial community structure and function to decrease GHG emissions and increase the yield of the summer maize-winter wheat rotation system.

## Introduction

The increase in yield is important for population growth and food security. However, it is not feasible to increase yield by increasing input (Cui et al., 2018; Meng et al., 2018). If the increase in yield is due to the increase of inputs without considering the environmental cost, these increased inputs will have a devastating impact on the environment, including greenhouse gas (GHG) emissions (Davidson, 2009; Burney et al., 2010), biodiversity loss (Tian et al., 2016), land degradation and freshwater pollution (Diaz and Rosenberg, 2008). There are many problems in northern China’s summer maize–winter wheat rotation system (MWRS), such as high investment, low fertilizer use efficiency, and serious resource waste. Therefore, reasonable agronomic management modes are important to improve the annual yield and fertilizer use efficiency of MWRS, which can ensure food security and protect the environment. Integrated Soil-Crop Management (ISCM) is an integrated management system designed to maximize the use of solar radiation and favorable temperatures and an effective crop management strategy based on “gene × environment × management” and the synchronization of inputs and supplies between soil, environment, and applications at a given site (Chen et al., 2011, 2014; Cui et al., 2018). In addition, the ISCM system is gradually expanded and some studies have confirmed the applicability of this system in the maize, rice, and wheat cropping system in China (Chen et al., 2014; Cui et al., 2018). The previous studies showed that the ISCM system can promote the growth and development of the aboveground parts of the crop, promote root development, improve crop dry matter accumulation, reduce soil N redundancy, and ultimately achieve a synergistic increase in yield and efficiency (Jin et al., 2012; Liu et al., 2017, 2018a,b; Yu et al., 2021, 2022). Our previous research has mostly focused on yield and N efficiency, but crop growth and yield formation cannot be achieved without changes in soil properties. In this study, we studied how changes in soil characteristics and microorganisms affect crop yield formation through ISCM long-term positioning experiments.

Soil microorganisms are the most reactive part of the soil ecosystem, driving soil biochemical processes with their own metabolism (Bernhard et al., 2007) and play an important role in soil carbon and nitrogen cycling and maintaining ecosystem stability (Canfield et al., 2010; Kuypers et al., 2018). The soil microbial community affects many ecosystem processes, including decomposition, nutrient mineralization, and plant nutrient acquisition and growth (Carney and Matson, 2005; Reeve et al., 2010; Talbot et al., 2014; You et al., 2014) and are sensitive to the soil environment changes (Brant et al., 2006). Agronomic management practices regulate the soil microbial community structure and function mainly by influencing soil pH, aggregate size and stability, moisture, organic carbon, and nutrient content (Hansel et al., 2008; Chu et al., 2010; Eilers et al., 2012). Fertilizer inputs, planting diversity, and tillage practices affect soil community structure and function in different ways (Mbuthia et al., 2015; Álvarez-Martín et al., 2016). Soil tillage regulates soil microbial community structure and diversity mainly by affecting soil physicochemical characteristics and microbial habitat (Somova and Pechurkin, 2001; Helgason et al., 2009). Fertilizer regulates soil microbial diversity, activity, quantity, and community structure to alter soil nutrient transformation and ultimately soil nutrient effectiveness (Cui et al., 2018; Ikoyi et al., 2020). Straw, crop roots, and root secretions provide carbon and energy sources for soil microorganisms (Zheng et al., 2015; Zhu et al., 2015; Wang et al., 2019). The ISCM systematically integrated management practices such as tillage practices, straw utilization, planting density, sowing date, fertilizer application and application period, and harvest date. The effects of single agronomic management on soil microbiota have been widely studied. However, how ISCM affects microbial structure and function through soil physicochemical properties and the response of yields and GHG emissions to microbial changes has remained poorly understood. Therefore, it is important to study the mechanisms of ISCM affecting soil quality, yield, and nitrogen use efficiency by investigating soil microorganisms.

Long-term field experiments would provide valuable information for the sustainability of intensive agriculture. In this study, we evaluated the contribution of changes in soil bacterial communities and soil physicochemical characteristics to the annual yield, nitrogen use efficiency, and GHG emission of MWRS based on a long-term ISCM experiment. This study was conducted to investigate (1) the effects of ISCM on soil characteristics, soil bacterial community distribution, and soil microbial functions; (2) the effects of ISCM on the correlations between microbes and environmental factors; and (3) the contributions of soil physicochemical factors and microorganisms to the increase of yield and nitrogen use efficiency and the decrease of GHG emission.

## Materials and methods

### Plant materials and experimental design

A long-term experimental field was established in 2009 at Da Wenkou Town, Tai’an, China (Supplementary Figure S1). The soil samples were obtained in 2018. The soil is classified as Eutriccambisols (WRB, 2015). This region is a temperate continental monsoon climate. Crops were planted twice a year. Summer maize (“Zhengdan 958,” ZD958) and winter wheat (“Tainong 18,” TN18) were used for experimental material. The treatments were used in a randomized block test design with four replicates, where each plot covered an area of 240 m^2^, 40.0 m long, and 6.0 m wide.

The field experiments were conducted using the ISCM. In the ISCM, the T1, T2, T3, and T4 methods integrated the cultivation method, planting density, fertilizer application management, and harvest time. The T1 was conducted according to the plant density and fertilizer method of local farmers; winter wheat straw was covered on the ground, and summer maize was no-tillage before sowing. The T2 increased planting density for the two annual crops with straw–returning and rotary tilling, decreased nitrogen fertilizer rate, changed one fertilization to divide fertilization in maize season at jointing stage and tassel stage, increased phosphate fertilizer and potassium fertilizer in wheat season, and, finally, delayed maize harvesting by 10 days to create a higher fertilizer use efficiency and higher yield compared with T1. Based on the T2, we defined the T3 by further increasing the planting density and fertilizer rate for both of the annual crops, increasing nitrogen application time in maize season at the jointing stage, tassel stage, and 1 week after the tassel stage to satisfy the needs of crop growth at high density, and late harvest time to realize super high yield and explore the yield potential in the region, which maximized yields without regard to costs and the fertilizer use efficiency was decreased. Finally, based on the T3, T4 decreased the maize planting density, increased the wheat planting density, decreased the fertilizer rate, and optimized fertilizer time to create synergistic improvements in nitrogen use efficiency and yield. Nitrogen fertilizer (urea, containing 46% N), phosphate fertilizer (calcium superphosphate, containing 17% P_2_O_5_), and potassium fertilizer (potassium chloride, containing 60% K_2_O) were applied to the test. The combination details of the tillage method, plant density, seeding, harvest dates, and fertilizer application are listed in Table 1.

**Table 1:** Cultivation management and fertilizer strategies of different integrated soil-crop managements.

Crop	Treatment	Tillage method	Seeding rate (×10^4^ seeds ha^−1^)	*SD* (m/d)	*HD* (m/d)	The periods and rates (kg ha^−1^) of fertilizer application					
						F	TF	BS	JS	VT–M	VT + 1 W–M
Summer maize	T1	Straw-unreturning, direct seeding	6	6/15	9/20	N	225		225		
						P	45		45		
						K	75		75		
	T2	Straw-returning, deep tillage before seeding	6.75	6/15	10/1	N	160.5	45	115.5		
						P	45	45			
						K	75	45	30		
	T3	Straw-returning, deep tillage before seeding	8.7	6/15	10/4	N	450		135	225	90
						P	150	60	90		
						K	300	150	150		
	T4	Straw-returning, deep tillage before seeding	7.5	6/15	10/4	N	184.5	30	90	64.5	
						P	55.5	30	25.5		
						K	130.5	30	70.5	30	
Winter wheat	T1	Straw-returning, rotary tillage before seeding	225	9/25	6/15	N	315	157.5	157.5		
						P	120	120			
						K	30	30			
	T2	Straw-returning, rotary tillage before seeding	300	10/5	6/12	N	240	96	144		
						P	150	150			
						K	75	75			
	T3	Straw-returning, rotary tillage before seeding	375	10/5	6/12	N	315	94.5	220.5		
						P	180	180			
						K	120	120			
	T4	Straw-returning, rotary tillage before seeding	450	10/5	6/12	N	240	72	168		
						P	150	150			
						K	120	72	48		

Before sowing, T3 was treated with 30 kg ha^−1^ ZnSO_4_. SD, sowing date; HD, harvest date; F, fertilizer; TF, total fertilizer rate; BS, before seeding; JS, jointing stage; VT–M, tassel stage of summer maize; VT + 1 W–M, 1 week after tassel of summer maize.

### Soil sample collection

The 0–30 cm soil was collected by soil auger and was used to determine the soil nutrient content at the jointing stage of wheat, maturity stage of wheat, tasseling stage of maize, and maturity stage of maize using the five-point sampling method in 2018. The five parallel samples were collected for each treatment and were placed in the ice box, and then immediately transported to the laboratory. One part of the soil samples were frozen at −80°C to be used later for DNA extraction and the other was stored at 4°C to determine the soil characteristics.

### Soil characteristics

The soil pH (soil: water ratio of 1: 2.5) was determined using a pH electrode (Leici, Shanghai, China). The soil total organic carbon (TOC) was determined *via* hydration thermal potassium dichromate oxidation. The total nitrogen (TN), total phosphorus (TP), and total potassium (TK) content in the soil was determined after being treated by the H_2_SO_4_-H_2_O_2_ mixture. And TN and TP were measured by an AA3 continuous flow analyzer (AutoAnalyzer 3, SEAL Analytical GmbH, Norderstedt, Germany). The TK was analyzed by flame atomic spectrophotometry. Alkali-hydrolyzed nitrogen (AN) was measured by the diffusion method. Available phosphorus (AP) was extracted by the Olsen method and was measured by Mo-Sb colorimetric method. Available potassium (AK) was extracted by the ammonium acetate method and was analyzed by flame atomic spectrophotometry.

### DNA extraction and Illumina MiSeq sequencing

The EZNATM Soil DNA Kit (Omega, United States) was used to extract the DNA of the soil at a UV-sterilized ultraclean bench. We used 1% (m/v) agarose gel electrophoresis to analyze the eluted DNA samples and used a NanoDrop® ND-1000 UV–Vis spectrophotometer (Thermo Fisher Scientific, United States) to measure the DNA concentration. The V3-V4 hypervariable regions of the bacterial 16S rRNA gene were amplified using Liu et al. (2021) method with the primer (338F 5′- ACTCCTACGGGAGGCAGCAG-3′ and 806R 5′-GGACTACHVGGGTWTCTAAT-3′). Amplification was performed with predenaturation for 3 min at 95°C, followed by 26 cycles of 30 s at 95°C, 30 s at 55°C, and 45 s at 72°C, and final extension for 10 min at 72°C. PCR amplicons were detected by electrophoresis on 2% (w/v) gels, and the Axygen® AxyPrep DNA Gel Extraction Kit (Axygen Biosciences, United States) was used to recover the target fragments. The purified amplicons were sequenced on the Illumina MiSeq PE250 sequencer.

### Analysis of the sequencing data

The high-throughput data were preliminarily processed using the method of Liu et al. (2021). And 8,718 OTUs were obtained after clustering after quality control and filtration. The sequence number of different samples was normalized to 25,454 (the sequence number of the fewest sequence) to accurately assess the diversity of the microbial communities. In this study, the subsequent analyzes were based on normalized data. The Shannon-Wiener and rarefaction curves (Supplementary Figure S3) eventually leveled off, which indicated that the sequencing depth was sufficient for subsequent data analysis.

### Soil greenhouse gas (GHG) emissions

The detailed N_2_O, CH_4_, and CO_2_ flux measurement procedures are reported in Li et al. (2013) using static chamber gas chromatography (40.0 cm wide, 40.0 cm long, and 20 cm high) with 3 replications. The GHG emissions were measured on the first day after sowing and fertilization subsequent sampling every other day, with measurement performed 1 week later. Adjustments to the sampling dates and frequency were made to include specific events, such as heavy rainfall and fertilization. Forty milliliters of gas were removed from the chamber to a sealed syringe after sealing the chamber at 0, 10, 20, and 30 min between 8:00–11:00 AM. N_2_O, CH_4_, and CO_2_ concentrations were determined by an Agilent GC7890 gas chromatographer (Agilent, California, United States).

### Statistical analysis

The *α*-Diversity (including Shannon, Simpson, Chao 1, and Ace) was calculated by Mothur software^1^ and *β*-diversity was calculated by QIIME software.^2^ Redundancy analysis (RDA) was performed using CANOCO5 software (Microcomputer Power, Ithaca, United States). The LEfSe tool in the online Galaxy/Hutlab^3^ was used to identify the biomarkers in the different treatments. The psych package in R software was used to perform the co-occurrence network analysis and the Gephi software (g ephi:) was used to perform the visualization of network relationships. The Hmisc package in R software was used to calculate Spearman correlations of soil characteristics, yield, nitrogen use efficiency, GHG emission, and microbial diversity. The TBtools was used to draw the correlation heatmap.^4^ The C and N cycle-related functions from the bacterial community were predicted using the FAPROTAX (derived from Functional Annotation of Prokaryotic Taxa) database (Louca et al., 2016), which is more suitable for functional annotation prediction of biogeochemical cycling processes (especially carbon, nitrogen, phosphorus, sulfur, and other elemental cycles). Louca et al. (2016) wrote a set of python scripts linking the OTU classification table with the FAPROTAX database, and the 16S-based OTU classification table can be simply passed through the python scripts to output the microbial community function annotation prediction results. The structural equation model (SEM) was used to link the change in soil microorganisms to soil characteristics, GHG emission, and summer maize-winter wheat yield and nitrogen using AMOS software (IBM SPSS Amos 23, SPSS Inc., Chicago, IL, United States) by generalized least squares (GLS) estimation. A one-way ANOVA (SPSS 16.0, SPSS Inc., Chicago, IL, United States) was used to analyze the differences in yield, GHG emissions, and soil characteristics. Significance was determined by Duncan’s test at a 0.05 significance level. The bar and line graphs were plotted using Sigma Plot 12.5. The R software^5^ was used to draw the boxplots.

## Results

### Soil characteristics

**Figure 1:**
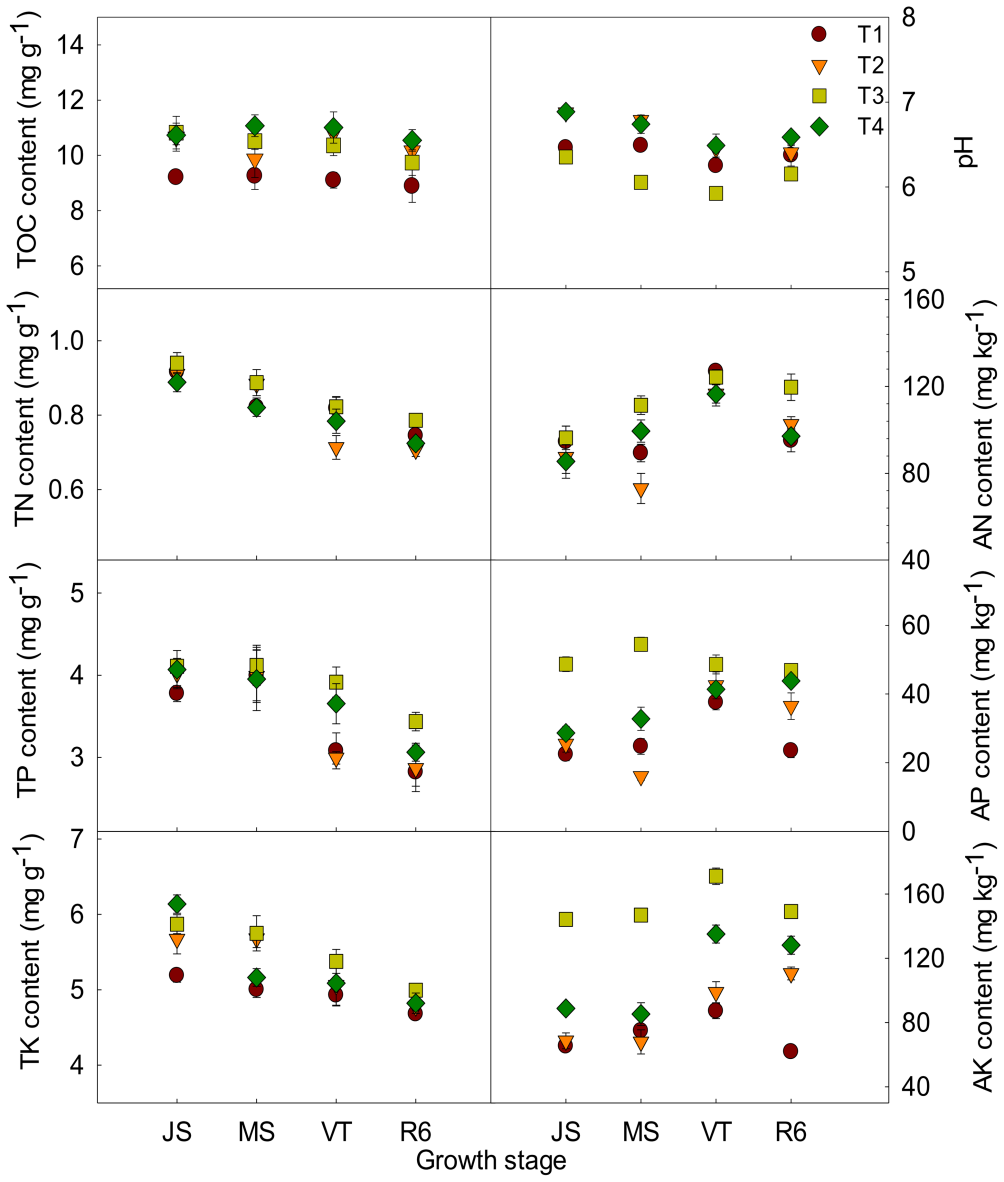
Effects of integrated soil-crop management on soil characteristics of summer maize-winter wheat. JS, Jointing stage of wheat; MS, maturity stage of wheat; VT, tasseling stage of maize; R6, maturity stage of maize; TOC, soil total organic carbon; TN, total nitrogen; AN, alkali-hydrolyzed nitrogen; TP, total phosphorus, AP, available phosphorus, TK, total potassium, AK, available potassium.

The ISCM could affect soil characteristics (Figure 1). The TOC content of T2, T3, and T4 was increased compared to that of T1. Take the MS as an example, the TOC content of T2, T3, and T4 was increased by 6.57, 13.61, and 19.59% compared to that of T1, respectively. The pH of T2 and T4 was increased after decreasing nitrogen rate compared with that of T1. However, the large fertilizer of T3 resulted in soil acidification (Figure 1). At MS, the pH of T2 and T4 was increased 4.60 and 3.88% compared to that of T1, respectively, and that of T3 was reduced by 6.69%. The content of TN, TP, and TK was increased with the increase of fertilizer input and those of T3 were the highest among the four treatments (Figure 1). The AP content and AK content of T2 and T4 were increased compared to that of T1. Take the tasseling stage of maize as an example, AP and AK of T2 and T4 were increased by13.31, 10.38 and 13.47%, 55.04% compared to that of T1, respectively. There was no significant difference in AN content between T4 and T1 (Figure 1).

### Bacterial *α*- and *β*-diversity

The Shannon and 1/simpson indexes showed that the changes in bacterial *α*-diversity were significant at R6 and were not significant at other stages (Supplementary Figure S4). The principal coordinate analysis (PCoA) showed differences in bacterial *β*-diversity at OTU level (Supplementary Figure S5). The two main coordinates of PCoA together explained the variation by 85.31, 82.13, 77.74, and 73.37% in bacterial *β*-diversity at the jointing stage of wheat (JS), maturity stage of wheat (MS), tasseling stage of maize (VT), and maturity stage of maize (R6), respectively. The OTUs of MS, VT, and R6 were separated, indicating that the ISCM could affect the bacterial community significantly (Adnois, *p* < 0.05).

### Bacterial community composition

At the phylum level, Acidobacteria (37.7%) was clearly dominant, followed by Proteobacteria (16.7%), Chloroflexi (15.8%), Actinobacteria (9.2%), Nitrospirae (4.7%), Gemmatimonadetes (4.6%), Firmicutes (2.6%), Bacteroidetes (1.6%), Planctomycetes (1.6%), Latescibacteria (1.5%), and Saccharibacteria (1.3%; Supplementary Figure S6). From phylum to genus, we identified ten biomarkers that showed significant differences in abundance among the four growth stages of the ISCM treatments (Supplementary Figure S7). At the four growth stages, five dominant species were enriched in T2 and T4 (*p* < 0.05) significantly, such as Acidobacteria (class), OM190 (class), Erysipelotrichaceae (family), *Polycyclovorans* (genus), and Anaerolineaceae (family). Five dominant species were enriched in T3 significantly, including Frankiales (order), Gaiellales (order), Caulobacteraceae (order), Elev-16S-1,332 (family), and *Rhodanobacter* (genus). And four dominant species were enriched in T1 significantly, including OM190 (class), Anaerolineaceae (family), Erysipelotrichaceae (family), and *Polycyclovorans* (genus).

### The co-occurrence network

**Figure 2:**
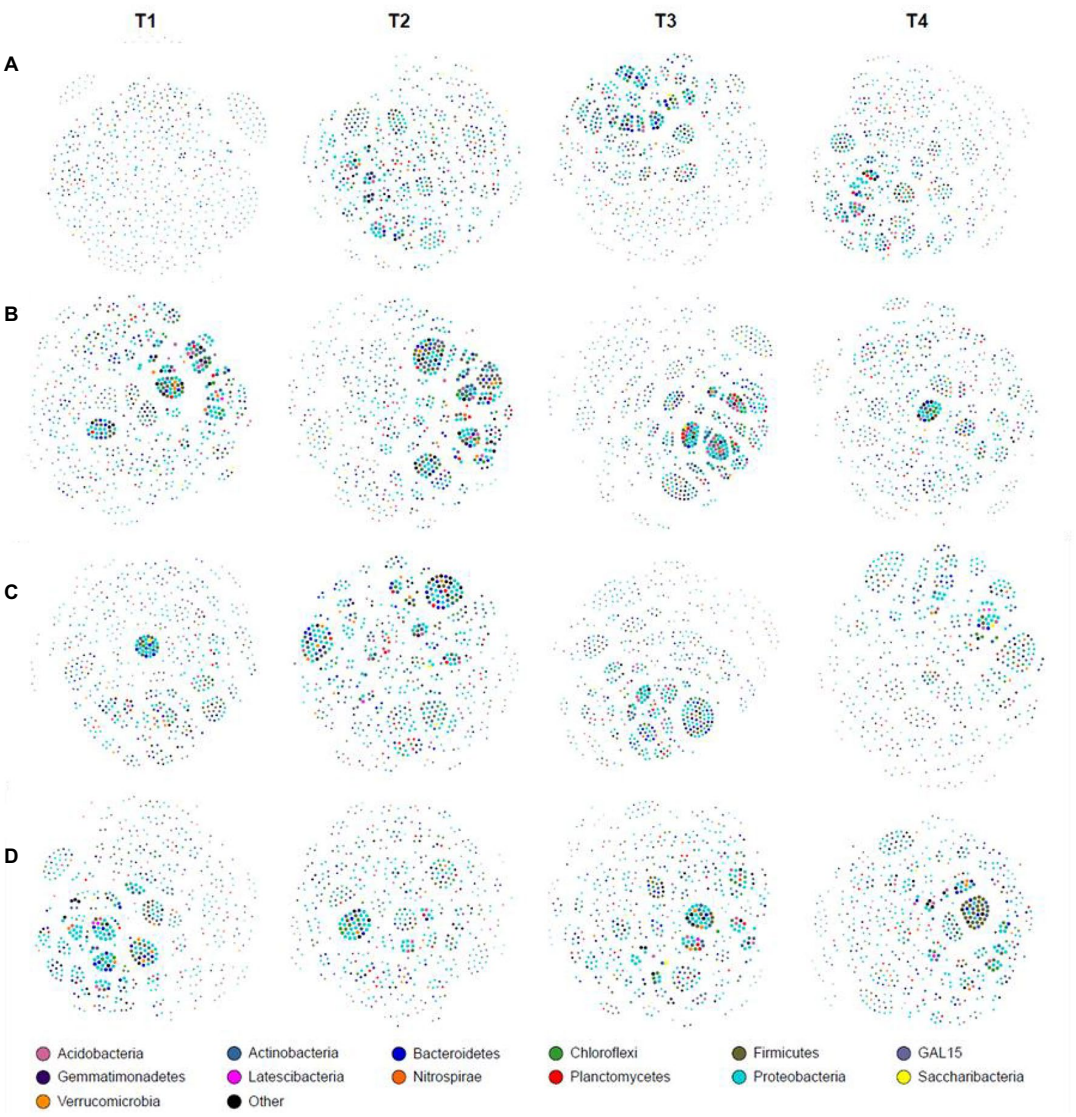
Co-occurrence network analysis in integrated soil-crop management. **(A)** Jointing stage of wheat; **(B)** maturity stage of wheat; **(C)** tasseling stage of maize; **(D)** maturity stage of wheat.

At the genus level, co-occurrence network analysis was different in different growth stages (Figure 2). At the JS stage, the network density, number of edges, and average degree of T1 were the lowest among the four treatments. Optimized ISCM could increase the network density of microorganisms in descending order as T3 (0.257) > T4 (0.253) > T2 (0.235; Figure 2A and Supplementary Table S1). At the MS stage, the network density of T3 was highest, 0.310, followed by T2 (0.263), T1 (0.241), and T4 (0.224); the number of edges and average degree showed the same trend (Figure 2B and Supplementary Table S1). At the VT stage, the network density among the four treatments was more than 0.240, which were 0.264 (T1), 0.247 (T2), 0.293 (T3), and 0.254 (T4) respectively, but the proportion of positive edges and average clustering coefficient of optimized ISCM were increased, which showed that T3 > T4 > T2 > T1 (Figure 2C and Supplementary Table S1). At the R6 stage, the network density, number of edges, and average degree of T1 were the highest among the four treatments (Figure 2D and Supplementary Table S1).

### Functional characteristics of microorganisms

**Figure 3:**
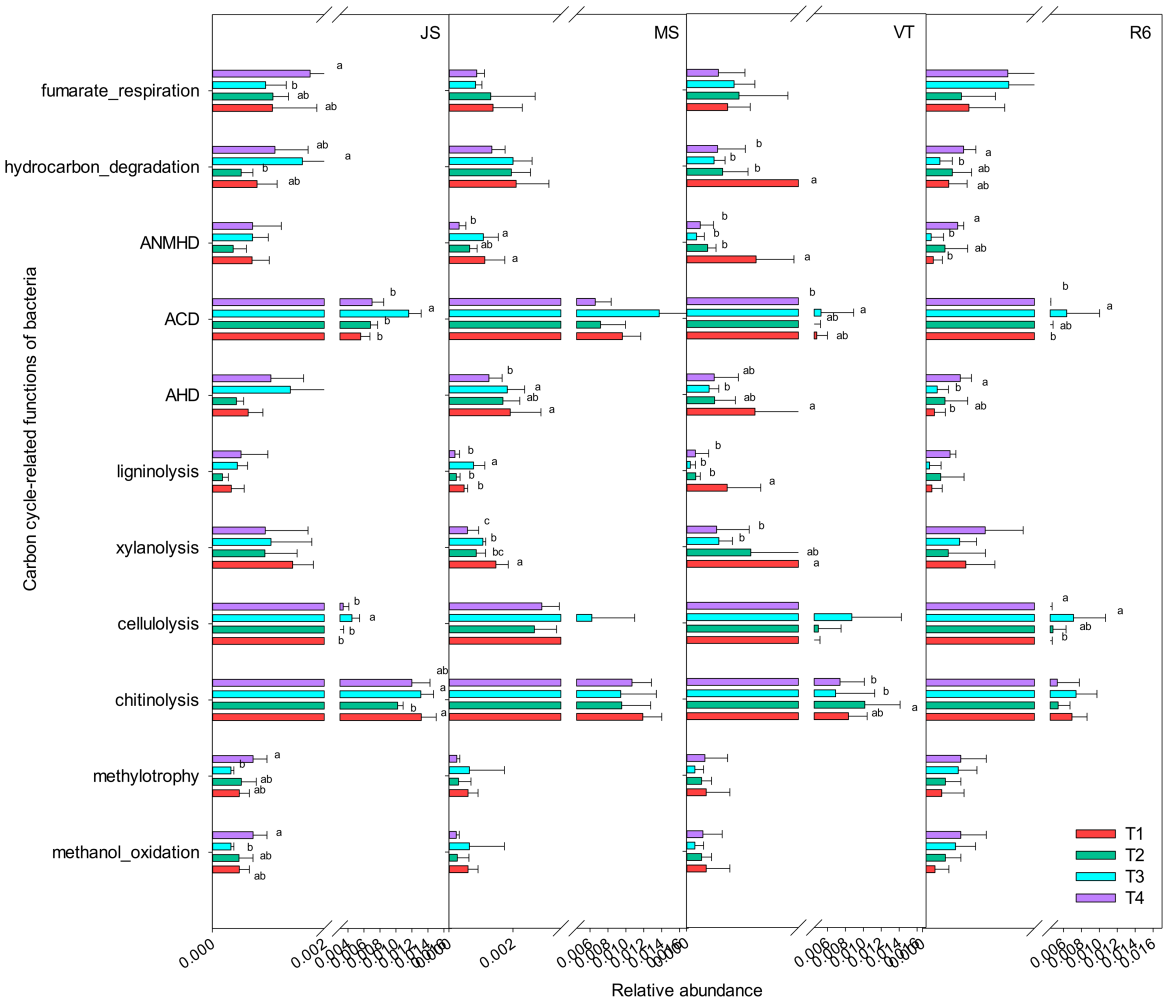
Carbon cycle-related functions of the bacterial communities as predicted by FAPROTAX. AHD, aromatic hydrocarbon degradation; ACD, aromatic compound degradation; ANMHD, aliphatic non-methane hydrocarbon degradation. Different letters represent significant differences (*p* < 0.05). JS, jointing stage of wheat; MS, maturity stage of wheat; VT, tasseling stage of maize; R6, maturity stage of wheat.

A total of 13 carbon cycle-related functional groups were annotated, such as xylanolysis, fumarate respiration, cellulolysis, aromatic compound degradation, and chitinolysis (Figure 3). The relative abundance of methylotrophy, methanol oxidation, aromatic hydrocarbon degradation, fumarate respiration, and hydrocarbon degradation of T4 treatment were significantly increased at the JS stage, compared to T1 (*p* < 0.05; Figure 3). The relative abundance of aliphatic non-methane hydrocarbon degradation, aromatic hydrocarbon degradation, hydrocarbon degradation, and xylanolysis of T1 treatment were significantly increased at MS and VT stages, compared to T4. At R6, the relative abundance of aromatic hydrocarbon degradation and hydrocarbon degradation of T4 treatment was significantly increased compared with that of T1.

**Figure 4:**
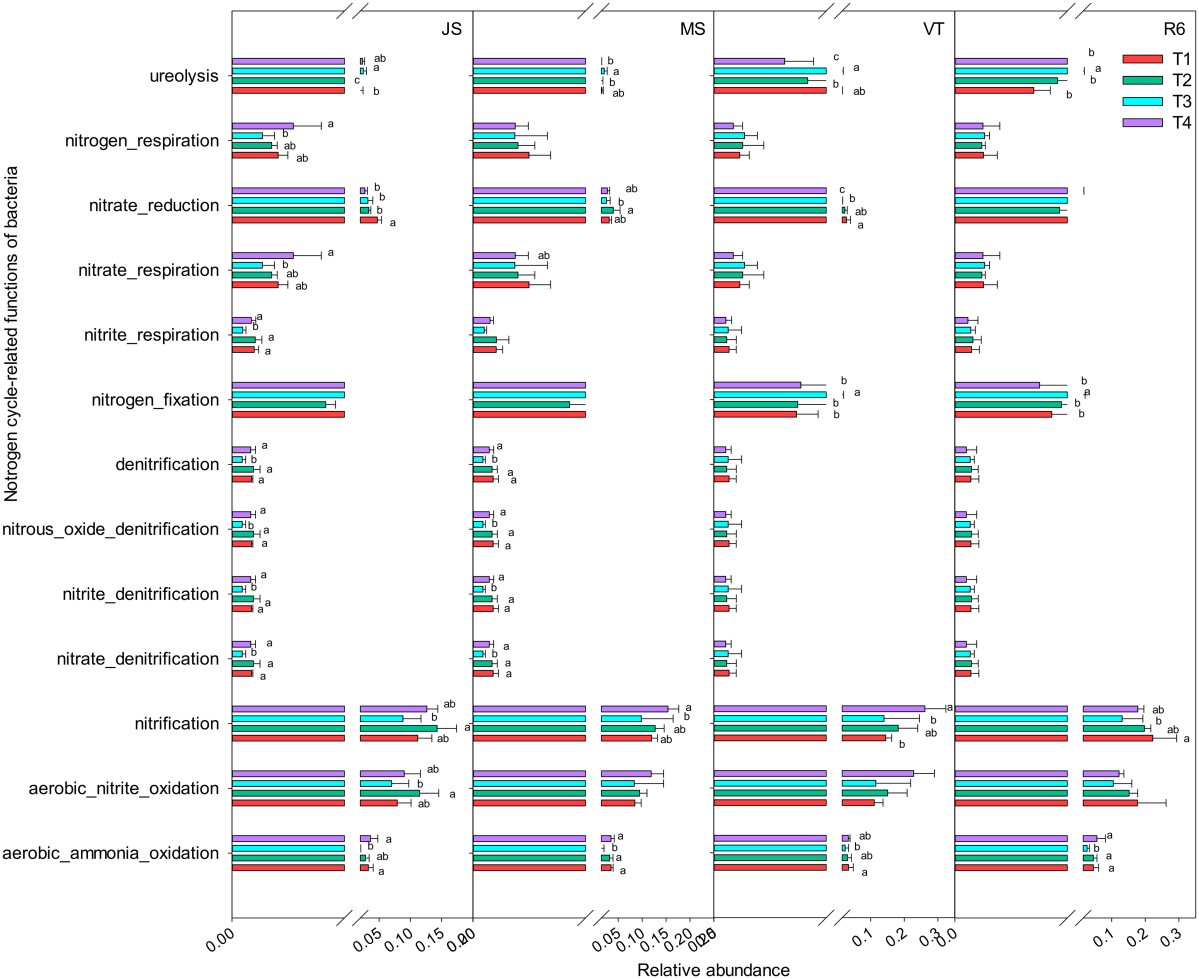
Nitrogen cycle-related functions of the bacterial communities as predicted by FAPROTAX. Different letters represent significant differences (*p* < 0.05). JS, jointing stage of wheat; MS, maturity stage of wheat; VT, tasseling stage of maize; R6, maturity stage of wheat.

A total of 15 nitrogen cycle-related functional groups were annotated, including aerobic nitrite oxidation, nitrification, aerobic ammonia oxidation, nitrate reduction, nitrogen respiration, and ureolysis (Figure 4). At JS and MS stages, the relative abundance of nitrogen cycle-related functional groups under T3 treatment was decreased compared to that of T4 and T1. However, T3 treatment increased the relative abundance of ureolysis and nitrogen fixation at VT and R6 (Figure 4). At VT, T4 increased the relative abundance of aerobic nitrite oxidation and nitrification and T1 increased the relative abundance of nitrate reduction and ureolysis among the four treatments. At other growth stages, T4 treatment and T1 treatment showed similar patterns in most nitrogen-related functional groups (Figure 4).

### The correlations between environmental factors and microorganisms

**Figure 5:**
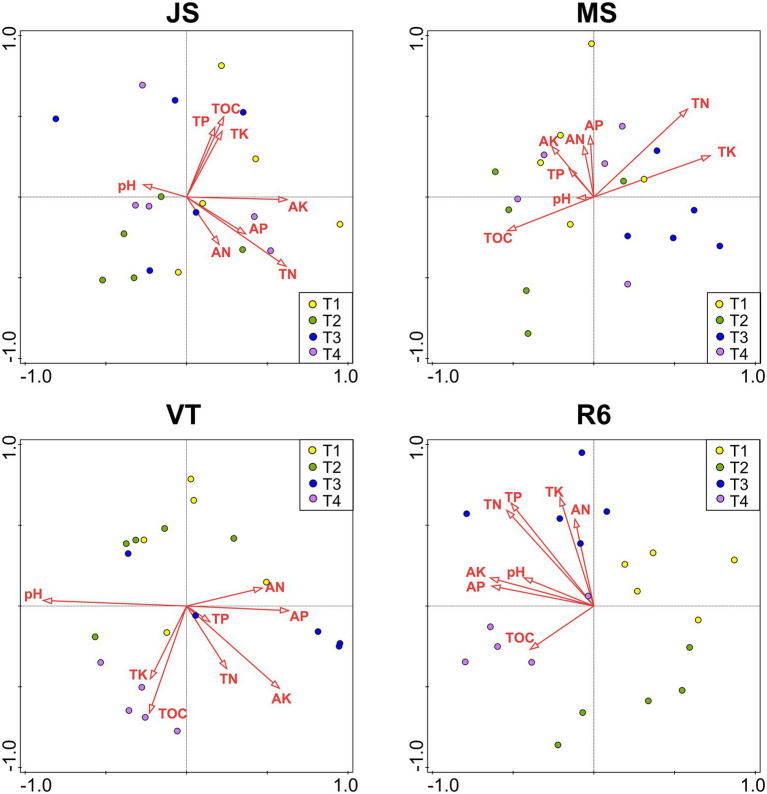
RDAs between the bacterial community of all OTUs with soil characteristics. JS, jointing stage of wheat; MS, maturity stage of wheat; VT, tasseling stage of maize; R6, maturity stage of wheat; TOC, soil total organic carbon; TN, total nitrogen; AN, alkali-hydrolyzed nitrogen; TP, total phosphorus, AP, available phosphorus, TK, total potassium, AK, available potassium.

At JS, the RDA explained the variation by 42.2% of the soil bacterial community (Figure 5). The AK (explanation rate = 9.4%) had the greatest effect on soil bacteria, followed by AP, pH, TK, and TOC. At MS, the RDA explained the variation by 48.6% of the soil bacterial community. The TN (explanation rate = 9.6%) had the greatest effect on the soil bacteria, followed by pH, TOC, and AP. The RDA explained the variation by 61.5% of the soil bacterial community at VT. The pH, TN, AP, and TOC significantly affected the soil bacterial community (*p* < 0.05). At R6, the explanation rate of RDA was 52.2%. AN, AK, and TK had great effects on the bacterial community structure, and the explanation rates were 12.6%, 12.45, and 10.8%, respectively, followed by TN.

**Figure 6:**
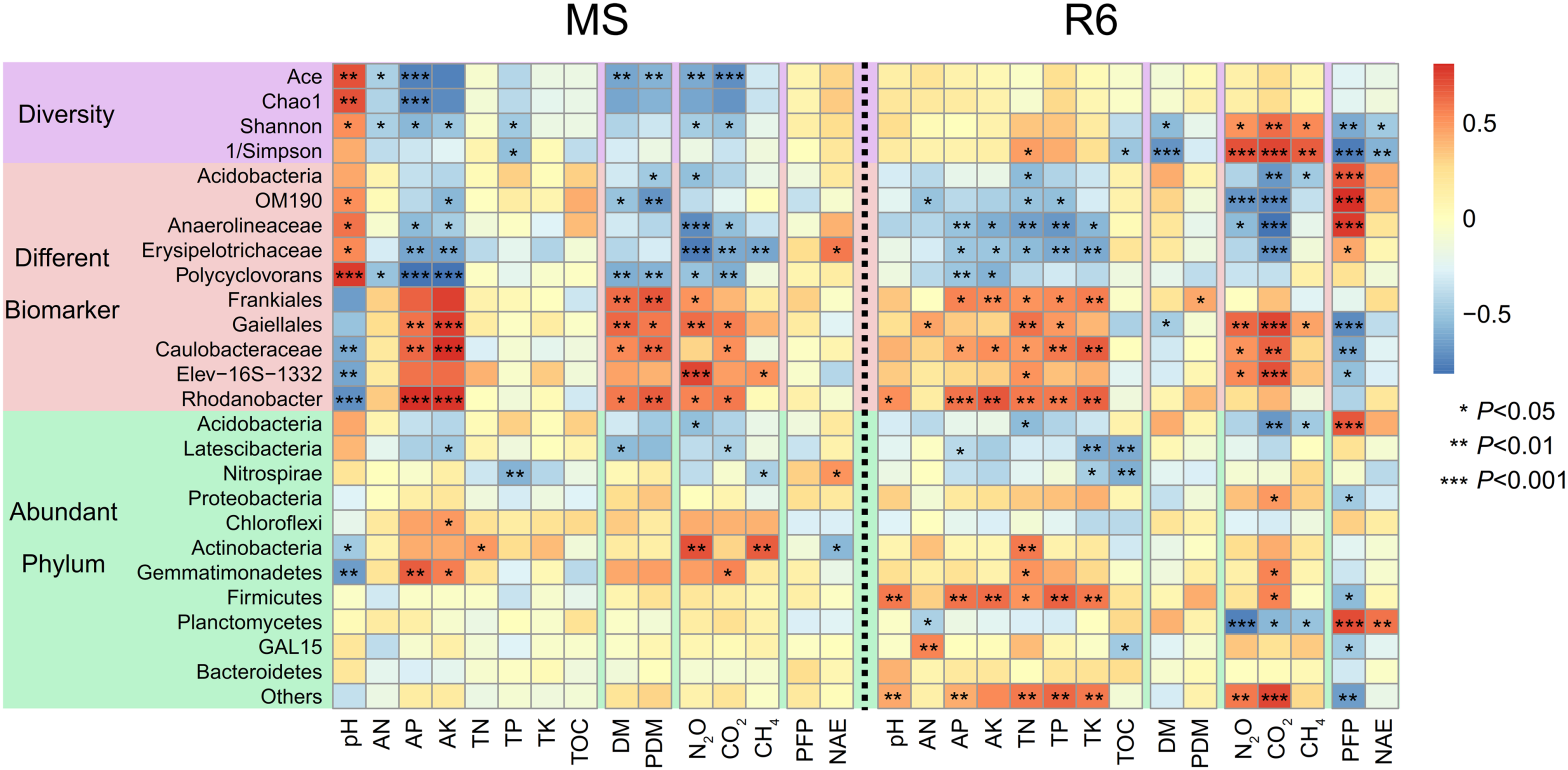
Spearman correlation heat maps of environmental factors and bacterial diversity, different biomarkers, and abundant phylum. Red represents a positive correlation, and blue represents a negative correlation (^*^p < 0.05; ^**^p < 0.01; ^***^p < 0.001). MS, maturity stage of wheat; R6, maturity stage of wheat; DM, dry matter of crop; PDM, population dry matter of crop; PFP, N partial factor productivity; NAE, N agronomic efficiency; TOC, soil total organic carbon; TN, total nitrogen; AN, alkali-hydrolyzed nitrogen; TP, total phosphorus, AP, available phosphorus, TK, total potassium, AK, available potassium.

Spearman correlation analyzes showed that the pH and AP were significantly positively correlated with the Shannon, Chao1, and Ace indexes at MS; the AN was significantly positively correlated with the Shannon indexes; the Ace index was significantly negatively correlated with dry matter accumulation and GHG emission; most of the different biomarker showed significant correlation with pH, AK, AP, dry matter accumulation, and GHG emission; Gemmatimonadetes were significantly negatively correlated with pH and significantly positively correlated with AP and AK (Figure 6). At R6, bacterial diversity, Shannon, and 1/Simpson indexes were significantly positively correlated with GHG emission and significantly negatively correlated with nitrogen use efficiency; most of the different biomarkers showed significant correlation with soil characteristics, GHG emission, and PFP_N_; Firmicutes were significant correlation with soil characteristics; Acidobacteria and Gemmatimonadetes were significantly positively correlated with TN; Planctomycetes were significantly positively correlated with nitrogen use efficiency and significantly negatively correlated with GHG emission (Figure 6).

### The structural equation model (SEM)

**Figure 7:**
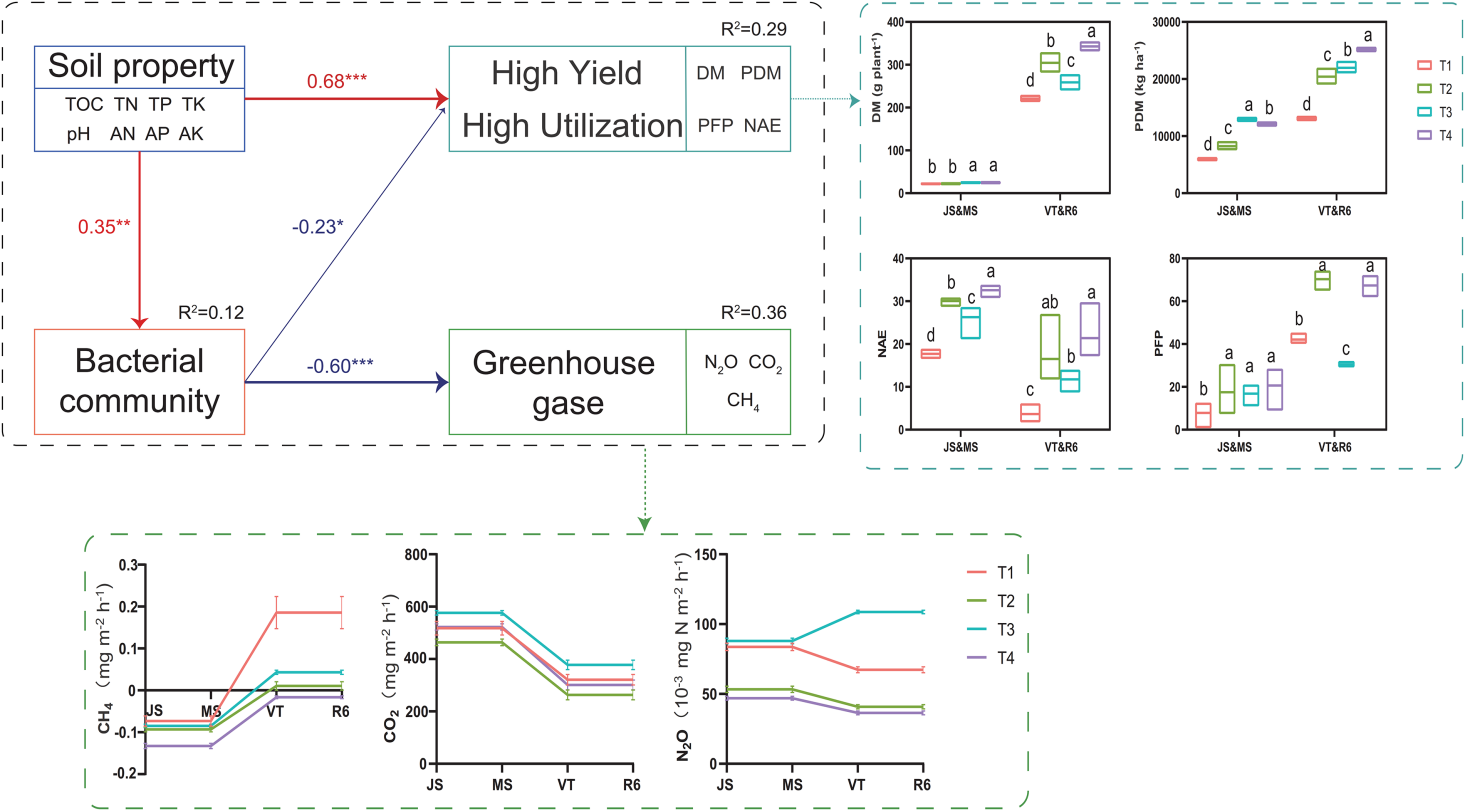
The value above the structural equation model (SEM) line represents the path coefficient. The red line represents the positive path coefficient, and the blue line represents the negative path coefficient ^(***^p < 0.001; ^**^p < 0.01; ^*^p < 0.05). JS, jointing stage of wheat; MS, maturity stage of wheat; VT, tasseling stage of maize; R6, maturity stage of wheat; DM, dry matter of crop; PDM, population dry matter of crop; PFP, N partial factor productivity; NAE, N agronomic efficiency.

SEM analyzed the effect of ISCM on soil characteristics, soil bacteria, GHG emission, nitrogen use efficiency, and annual yield, which explained 29% of the variation in nitrogen use efficiency and summer maize-winter wheat annual yield, 12% of the variation in soil bacteria, and 36% of the variation in GHG emission (Figure 7). The N_2_O and CH_4_ emission of T4 significantly reduced compared to that of T1 (Figure 7 and Supplementary Figure S2). However, the N_2_O and CO_2_ emission of T3 was highest. The soil nutrient levels of ISCM treatments directly affected the annual yield and nitrogen use efficiency of summer maize-winter wheat (*λ*: 0.68, *p* < 0.001) and soil bacteria (*λ*: 0.35, *p* < 0.05) by changing the soil nutrient status. The influence of soil nutrients on GHG emissions (*λ*: −0.60, *p* < 0.001) was indirectly mediated by soil bacteria (*λ*: −0.35, *p* < 0.01).

## Discussion

### ISCM improves the soil nutrition

Soil physicochemical characteristics can evaluate soil quality. The tillage practices (Coulibaly et al., 2022), straw return (Huang et al., 2021), fertilization practices (Zhao et al., 2021), and cropping patterns (Gikonyo et al., 2022) can significantly affect soil physicochemical properties. Increasing the content of effective soil N, P and K can improve crop yield (Gondwe et al., 2020). Optimized integrated soil-crop management matches nutrient supply and crop demand quantitatively and synchronizes them temporally through regulating planting density and fertilization management to ensure fertilizer availability. Increasing the amount of available soil nitrogen, phosphorus and potassium can increase crop quality and yield (Gondwe et al., 2020). However, long-term over-fertilization can lead to soil acidification (Bhattacharyya et al., 2015), which was also confirmed in our study. However, the excessive fertilization of T3 resulted in a decrease in pH and soil acidification and an increase in GHG emissions (Supplementary Figure S2), which resulted in the reduction of nitrogen use efficiency. Optimized ISCM could increase pH by reducing fertilizer rate, increase the soil nutrient effectiveness, and decrease soil bulk density (Liu et al., 2017). The T3 treatment had the largest amount of straw returned to the field, but soil TOC did not increase proportionally and was significantly lower compared to that of the T4 treatment. This may also be related to the decrease in pH of T3 treatment. Higher soil pH is strongly associated with higher carbon utilization (Zhang et al., 2020). The lower pH of the T3 treatment may reduce carbon utilization (Zhang et al., 2020). And soil acidification leads to soil organic carbon loss and increases CO_2_ emissions from the soil (Jin and Wang, 2018). This is also one of the important reasons for the decrease in soil TOC and the increase in CO_2_ emission in T3 treatment. Optimized ISCM could increase soil TOC storage by returning straw in both summer maize-winter wheat seasons (Figure 1). Soil acidification and improved soil nutrition were reduced by optimizing ISCM, which increased the yield of double cropping of summer maize and winter wheat. This provides an environmentally preferable direction for sustainable production of soil.

### ISCM improves community composition and function of soil microorganisms

The ISCM could affect soil microorganism community composition and function, but the effect was different in the different crop growth stages. Optimized ISCM significantly enriched Acidobacteria (class), OM190 (class), Erysipelotrichaceae (family), and *Polycyclovorans* (genus). The Acidobacteria and the Aspergillus are abundant in soil (Hugenholtz et al., 1998; Barns et al., 1999) and are important members of the soil microorganisms, which have many genes encoding cellulases and hemicellulases to degrade plant residues (Kanokratana et al., 2011). They play an important role in soil material cycling and ecosystem construction (Eichorst et al., 2011; Pankratov et al., 2011). The enrichment of Acidobacteriad in T4 promoted the decomposition of straw (Supplementary Figure S6). Soil pH is an important factor in determining the structure of microbial communities and is the key driver to shaping microbial community structure, diversity, and composition (Jones et al., 2019). The lower pH of the T3 treatment may increase Al toxicity in the soil (Pietri and Brookes, 2008), which in turn affected some soil carbon cycling microbial activity, such as methanol oxidation and methylotrophy, and reduced biomass carbon conversion, resulting in lower soil TOC with high carbon input compared to the T4 treatment. In addition, the straw of winter wheat in T4 was returned to the field and the number of microorganisms related to the carbon cycle was increased (Figure 3), which further promoted the decomposition of straw in the soil and contributed to the increase in soil organic carbon content. The Actinomycetes can degrade a large number of organic compounds, which be significantly affected by soil pH (Fierer et al., 2014). In contrast, the enrichment of Frankiales and some bacilli in the T3 treatment (Supplementary Figure S6) may be related to the reduction of soil pH due to long-term heavy fertilization and a large amount of straw returned to the field.

In addition, ISCM could significantly affect microbial network density and change with the crop growth stage (Figure 2), which was closely related to the agronomic management practices in each period. The microorganism network density of T3 was the highest among the four treatments (except for the R6), which may be related to the fact that heavy fertilization accelerated the growth of soil microorganisms (Siles et al., 2021), but too many microbes may lead to increased competition and increase more negative interactions between species (Ratzke et al., 2020). However, Giovannoni et al. (2014) showed that microorganisms tend to coexist in large numbers in low nutrient environments. The lower soil nutrient content of the T1 treatment led to an increase in microorganism network density compared with the T4 treatment (except for the JS; Figure 2). The nitrogen fertilizer rate of T4 was lower compared with T1, but the nitrogen cycle-related functional groups of T4 showed similar patterns to T1, which may be conducive to the effective use of nitrogen in the soil and improve nitrogen use efficiency.

### Soil improvement reduces greenhouse gas emissions

The ISCM directly affected maize yield and bacterial distribution by altering soil nutrient status (Figure 7). The different biomarkers were significantly associated with soil GHG emissions and nitrogen use efficiency (Figure 7). Soil microorganisms play a central role in GHG emissions, and their abundance and activity are important biological factors influencing soil GHG emissions (Akiyama et al., 2014; Lafuente et al., 2019). Microbial respiration is an important source of CO_2_ in soil; the CH_4_ and N_2_O production is mainly related to methanogenic bacteria and nitrifying and denitrifying bacteria, respectively (Chidthaisong and Watanabe, 1997; Mer and Roger, 2001). In addition, the soil N content is the main N source of N_2_O emissions, and changes in aerobic and anaerobic soil conditions promote N_2_O emissions (Du et al., 2021). Rhodococcus has an intact denitrification capacity, which promotes N_2_O production (Takeda et al., 2012). This study showed that microbial community structure and function were significantly associated with soil GHG emissions (Figure 6). The nitrogen fertilizer rate of T4 was significantly lower compared with that of T1. However, the microorganisms related to nitrogen transformation of the T4 and T1 treatments were analogous (Figure 4) and the Rhodococcus of T4 decreased by 64.1 and 82.6% compared to that of T1 at JS and VT, which could be the reason for the significant increase in nitrogen use efficiency and the significant decrease in N_2_O emissions in the T4 treatment. The Rhodococcus of T2 also decreased by 94.2 and 2.4% compared to that of T1. In contrast, the T3 treatment resulted in an increase in soil N content due to heavy fertilization and enrichment of Rhodococcus that was increased by 555.6 and 195.2% compared with that of T1, possibly responsible for a significant increase in soil N_2_O emissions and a decrease in nitrogen use efficiency. Stevens and Laughlin (1998) showed that increasing NO_3_^−^ content had a small effect on the denitrification rate, but significantly increased N_2_O emissions. Although denitrification of T3 treatment was reduced in the wheat season, the amount of applied N fertilizer was too high, and the NO_3_^−^ content in the soil increased significantly (Jin et al., 2012), which could be one of the reasons for the increase in N_2_O emissions.

## Conclusion

The soil acidification was reduced and soil nutrition was improved by optimizing ISCM. Optimized ISCM improved the structure and the co-occurrence network density of soil microorganisms at the vigorous period of crop growth, reduced the abundance of bacteria associated with GHG emissions, and increased the abundance of bacteria associated with carbon and nitrogen cycling. These changes in soil microorganisms could promote carbon and nitrogen cycling in the soil and indirectly contribute to the decrease in GHG emissions and the increase in nitrogen use efficiency and yield. That may provide an efficient soil and crop management of summer maize-winter wheat cropping systems to promote soil healthy development in the future. Setting new and suitable ISCMs according to local environmental conditions can help achieve promote soil sustainable development.

## Data availability statement

The datasets presented in this study can be found in online repositories. The data presented in the study are deposited in the NCB Irepository, accession number PRJNA887216.

## Author contributions

NY and JL participated in the design of the experiment and wrote the manuscript and data analyzes. BR, BZ, PL, and ZG participated in data analyzes and engaged in useful discussion and revised the manuscript. JZ developed the concepts, designed and supervised the study, and revised the manuscript. All authors contributed to the article and approved the submitted version.

## Funding

This research was financially supported by the National Natural Science Foundation of China (32172115), the Shandong Province Key Research and Development Program (2021LZGC014-2), and the China Agriculture System of MOF and MARA (CARS-02-21).

## Conflict of interest

The authors declare that the research was conducted in the absence of any commercial or financial relationships that could be construed as a potential conflict of interest.

## Publisher’s note

All claims expressed in this article are solely those of the authors and do not necessarily represent those of their affiliated organizations, or those of the publisher, the editors and the reviewers. Any product that may be evaluated in this article, or claim that may be made by its manufacturer, is not guaranteed or endorsed by the publisher.

## Footnotes

^1^ www.mothur

^2^ http://qiime

^3^ http://huttenhower.sph.harvard.edu/galaxy/

^4^ http://www.tbtools.com/

^5^ www.r-project.org

## References

[ref1] Akiyama H. Morimoto S. Tago K. Hoshino Y. T. Nagaoka K. Yamasaki M. (2014). Relationships between ammonia oxidizers and N_2_O and CH_4_ fluxes in agricultural fields with different soil types. Soil Sci. Plant Nutr. 60, 520–529. doi: 10.1080/00380768.2014.904206

[ref2] Álvarez-Martín A. Hilton S. L. Bending G. D. Rodríguez-Cruz M. S. Sánchez-Martín M. J. (2016). Changes in activity and structure of the soil microbial community after application of azoxystrobin or pirimicarb and an organic amendment to an agricultural soil. Appl. Soil Ecol. 106, 47–57. doi: 10.1016/j.apsoil.2016.05.005

[ref3] Barns S. M. Takala S. L. Kuske C. R. (1999). Wide distribution and diversity of members of the bacterial kingdom acidobacterium in the environment. Appl. Environ. Microb. 65, 1731–1737. doi: 10.1128/AEM.65.4.1731-1737.1999, PMID: 10103274 PMC91244

[ref4] Bernhard A. E. Tucker J. Giblin A. E. Stahl D. A. (2007). Functionally distinct communities of ammonia-oxidizing bacteria along an estuarine salinity gradient. Envir. Mic. 9, 1439–1447. doi: 10.1111/j.1462-2920.2007.01260.x, PMID: 17504481

[ref5] Bhattacharyya R. Ghosh B. N. Mishra P. K. Mandal B. Rao C. S. Sarkar D. (2015). Soil degradation in India: challenges and potential solutions. Sustain 7, 3528–3570. doi: 10.3390/su7043528

[ref6] Brant J. B. Sulzman E. W. Myrold D. D. (2006). Microbial community utilization of added carbon substrates in response to long-term carbon input manipulation. Soil. Biol. Bioc. 38, 2219–2232. doi: 10.1016/j.soilbio.2006.01.022

[ref7] Burney J. A. Davis S. J. Lobell D. B. (2010). Greenhouse gas mitigation by agricultural intensification. Proc. Natl. Acad. Sci. 107, 12052–12057. doi: 10.1073/pnas.0914216107, PMID: 20551223 PMC2900707

[ref8] Canfield D. E. Glazer A. N. Falkowski P. G. (2010). The evolution and future of earth's nitrogen cycle. Science 330, 192–196. doi: 10.1126/science.1186120, PMID: 20929768

[ref9] Carney K. M. Matson P. A. (2005). Plant communities, soil microorganisms, and soil carbon cycling: does altering the world belowground matter to ecosystem functioning? Ecosystems 8, 928–940. doi: 10.1007/s10021-005-0047-0

[ref10] Chen X. P. Cui Z. L. Fan M. S. Vitousek P. Zhao M. Ma W. Q. (2014). Producing more grain with lower environmental costs. Nature 514, 486–489. doi: 10.1038/nature13609, PMID: 25186728

[ref11] Chen X. P. Cui Z. L. Vitousek P. M. Cassman K. G. Matson P. A. Bai J. S. (2011). Integrated soil–crop system management for food security. Proc. Nat. Acad. Sci. 108, 6399–6404. doi: 10.1073/pnas.1101419108, PMID: 21444818 PMC3080987

[ref12] Chidthaisong A. Watanabe I. (1997). Methane formation and emission from flooded rice soil incorporated with ^13^C–labeled rice straw. Soil Biol. Biochem. 29, 1173–1181. doi: 10.1016/S0038-0717(97)00034-5

[ref13] Chu H. Fierer N. Lauber C. L. Caporaso J. G. Knight R. Grogan P. (2010). Soil bacterial diversity in the Arctic is not fundamentally different from that found in other biomes. Environ. Microbiol. 12, 2998–3006. doi: 10.1111/j.1462-2920.2010.02277.x 20561020

[ref14] Coulibaly S. F. Aubert M. Brunet N. Bureau F. Legras M. Chauvat M. (2022). Short-term dynamic responses of soil properties and soil fauna under contrasting tillage systems. Soil Till. Res. 215:105191. doi: 10.1016/j.still.2021.105191

[ref15] Cui Z. L. Zhang H. Y. Chen X. P. Zhang C. C. Ma W. Q. Huang C. D. (2018). Pursuing sustainable productivity with millions of smallholder farmers. Nature 555, 363–366. doi: 10.1038/nature25785, PMID: 29513654

[ref16] Davidson E. A. (2009). The contribution of manure and fertilizer nitrogen to atmospheric nitrous oxide since 1860. Nat. Geosci. 2, 659–662. doi: 10.1038/ngeo608

[ref17] Diaz R. J. Rosenberg R. (2008). Spreading dead zones and consequences for marine ecosystems. Science 321, 926–929. doi: 10.1126/science.1156401, PMID: 18703733

[ref18] Du Y. G. Ke X. Li J. M. Wang Y. Y. Cao G. M. Guo X. W. (2021). Nitrogen deposition increases global grassland N_2_O emission rates steeply: a meta-analysis. Catena 199:105105. doi: 10.1016/j.catena.2020.105105

[ref19] Eichorst S. A. Kuske C. R. Schmidt T. M. (2011). Influence of plant polymers on the distribution and cultivation of bacteria in the phylum Acidobacteria. Appl. Environ. Microb. 77, 586–596. doi: 10.1128/AEM.01080-10, PMID: 21097594 PMC3020536

[ref20] Eilers K. G. Debenport S. Anderson S. Fierer N. (2012). Digging deeper to find unique microbial communities: the strong effect of depth on the structure of bacterial and archaeal communities in soil. Soil Biol. Biochem. 50, 58–65. doi: 10.1016/j.soilbio.2012.03.011

[ref21] Fierer N. Barberán A. Laughlin D. C. (2014). Seeing the forest for the genes: using metagenomics to infer the aggregated traits of microbial communities. Front. Microbiol. 5:614. doi: 10.3389/fmicb.2014.00614, PMID: 25429288 PMC4228856

[ref22] Gikonyo F. N. Dong X. Mosongo P. S. Guo K. Liu X. (2022). Long-term impacts of different cropping patterns on soil physico-chemical properties and enzyme activities in the low land plain of North China. Agronomy 12:471. doi: 10.3390/agronomy12020471

[ref23] Giovannoni S. J. Thrash J. C. Temperton B. (2014). Implications of streamlining theory for microbial ecology. ISME J. 8, 1553–1565. doi: 10.1038/ismej.2014.60, PMID: 24739623 PMC4817614

[ref24] Gondwe R. L. Kinoshita R. Suminoe T. Aiuchi D. Palta J. P. Tani M. (2020). Available soil nutrients and NPK application impacts on yield, quality, and nutrient composition of potatoes growing during the main season in Japan. Am. J. Potato Res. 97, 234–245. doi: 10.1007/s12230-020-09776-2

[ref25] Hansel C. M. Fendorf S. Jardine P. M. Francis C. A. (2008). Changes in bacterial and archaeal community structure and functional diversity along a geochemically variable soil profile. Appl. Environ. Microbiol. 74, 1620–1633. doi: 10.1128/AEM.01787-07, PMID: 18192411 PMC2258623

[ref26] Helgason B. L. Walley F. L. Germida J. J. (2009). Fungal and bacterial abundance in long-term no-till and intensive-till soils of the northern great plains. Soil Sci. Soc. Am. J. 73, 120–127. doi: 10.2136/sssaj2007.0392

[ref27] Huang T. Yang N. Lu C. Qin X. Siddique K. H. (2021). Soil organic carbon, total nitrogen, available nutrients, and yield under different straw returning methods. Soil Till. Res. 214:105171. doi: 10.1016/j.still.2021.105171

[ref28] Hugenholtz P. Goebel B. M. Pace N. R. (1998). Impact of culture-independent studies on the emerging phylogenetic view of bacterial diversity. J. Bacteriol. 180, 4765–4774. doi: 10.1128/JB.180.18.4765-4774.1998, PMID: 9733676 PMC107498

[ref29] Ikoyi I. Egeter B. Chaves C. Ahmed M. Fowler A. Schmalenberger A. (2020). Responses of soil microbiota and nematodes to application of organic and inorganic fertilizers in grassland columns. Biol. Fertil. Soils 56, 647–662. doi: 10.1007/s00374-020-01440-5

[ref30] Jin L. B. Cui H. Y. Li B. Zhang J. W. Dong S. T. Liu P. (2012). Effects of integrated agronomic management practices on yield and nitrogen efficiency of summer maize in North China. Field Crops Res. 134, 30–35. doi: 10.1016/j.fcr.2012.04.008

[ref31] Jin S. Wang H. (2018). Relationships between soil pH and soil carbon in China’s carbonate soils. Fresenius Environ. Bull. 27, 605–611.

[ref32] Jones D. L. Cooledge E. C. Hoyle F. C. Griffiths R. I. Murphy D. V. (2019). pH and ex-changeable aluminum are major regulators of microbial energy flow and carbon use efficiency in soil microbial communities. Soil Biol. Biochem. 138:107584. doi: 10.1016/j.soilbio.2019.107584

[ref33] Kanokratana P. Uengwetwanit T. Rattanachomsri U. Bunterngsook B. Nimchua T. Tangphatsornruang S. (2011). Insights into the phylogeny and metabolic potential of a primary tropical peat swamp forest microbial community by metagenomic analysis. Microb. Ecol. 61, 518–528. doi: 10.1007/s00248-010-9766-7, PMID: 21057783

[ref34] Kuypers M. M. Marchant H. K. Kartal B. (2018). The microbial nitrogen–cycling network. Nat. Rev. Microbiol. 16, 263–276. doi: 10.1038/nrmicro.2018.9 29398704

[ref35] Lafuente A. Bowker M. A. Delgado-Baquerizo M. Durán J. Singh B. K. Maestre F. T. (2019). Global drivers of methane oxidation and denitrifying gene distribution in drylands. Glob. Ecol. Biogeogr. 28, 1230–1243. doi: 10.1111/geb.12928

[ref36] Li C. F. Zhang Z. S. Guo L. J. Cai M. L. Cao C. G. (2013). Emissions of CH_4_ and CO_2_ from double rice cropping systems under varying tillage and seeding methods. Atmos. Environ. 80, 438–444. doi: 10.1016/j.atmosenv.2013.08.027

[ref37] Liu Z. Gao J. Gao F. Dong S. T. Liu P. Zhao B. (2018a). Integrated agronomic practices management improve yield and nitrogen balance in double cropping of winter wheat–summer maize. Field Crops Res. 221, 196–206. doi: 10.1016/j.fcr.2018.03.001

[ref38] Liu Z. Gao J. Gao F. Liu P. Zhao B. Zhang J. W. (2018b). Photosynthetic characteristics and chloroplast ultrastructure of summer maize response to different nitrogen supplies. Front. Plant Sci. 9:576. doi: 10.3389/fpls.2018.00576, PMID: 29765387 PMC5938403

[ref39] Liu J. A. Shu A. P. Song W. F. Shi W. C. Li M. C. Zhang W. X. (2021). Long-term organic fertilizer substitution increases rice yield by improving soil properties and regulating soil bacteria. Geoderma 404:115287. doi: 10.1016/j.geoderma.2021.115287

[ref40] Liu Z. Zhu K. L. Dong S. T. Liu P. Zhao B. Zhang J. W. (2017). Effects of integrated agronomic practices management on root growth and development of summer maize. Eur. J. Agron. 84, 140–151. doi: 10.1016/j.eja.2016.12.006

[ref41] Louca S. Parfrey L. W. Doebeli M. (2016). Decoupling function and taxonomy in the global ocean microbiome. Science 353, 1272–1277. doi: 10.1126/science.aad8279, PMID: 27634532

[ref42] Mbuthia L. W. Acosta-Martinez V. DeBruyn J. Schaeffer S. Tyler D. Odoi E. (2015). Long term tillage, cover crop, and fertilization effects on microbial community structure, activity: implications for soil quality. Soil Biol. Biochem. 89, 24–34. doi: 10.1016/j.soilbio.2015.06.016

[ref43] Meng Q. F. Cui Z. L. Yang H. H. Zhang F. S. Chen X. P. (2018). Establishing high–yielding maize system for sustainable intensification in China. Adv. Agron. 148, 85–109. doi: 10.1016/bs.agron.2017.11.004

[ref44] Mer J. L. Roger P. (2001). Production, oxidation, emission and consumption of methane by soils: a review. Eur. J. Soil Bio. 37, 25–50. doi: 10.1016/S1164-5563(01)01067-6

[ref45] Pankratov T. A. Ivanova A. O. Dedysh S. N. Liesack W. (2011). Bacterial populations and environmental factors controlling cellulose degradation in an acidic sphagnum peat. Environ. Microbiol. 13, 1800–1814. doi: 10.1111/j.1462-2920.2011.02491.x, PMID: 21564458

[ref46] Pietri J. A. Brookes P. C. (2008). Relationships between soil pH and microbial properties in a UK arable soil. Soil Biol. Biochem. 40, 1856–1861. doi: 10.1016/j.soilbio.2008.03.020

[ref47] Ratzke C. Barrere J. Gore J. (2020). Strength of species interactions determines biodiversity and stability in microbial communities. Nat. Ecol. Evol. 4, 376–383. doi: 10.1038/s41559-020-1099-4, PMID: 32042124

[ref48] Reeve J. R. Schadt C. W. Carpenter-Boggs L. Kang S. Zhou J. Reganold J. P. (2010). Effects of soil type and farm management on soil ecological functional genes and microbial activities. ISME J. 4, 1099–1107. doi: 10.1038/ismej.2010.42, PMID: 20376100

[ref49] Siles J. A. García-Sánchez M. Gómez-Brandón M. (2021). Studying microbial communities through co-occurrence network analyses during processes of waste treatment and in organically amended soils: a review. Microorganisms 9:1165. doi: 10.3390/microorganisms9061165, PMID: 34071426 PMC8227910

[ref50] Somova L. A. Pechurkin N. S. (2001). Functional, regulatory and indicator features of microorganisms in man-made ecosystems. Adv. Space Res. 27, 1563–1570. doi: 10.1016/S0273-1177(01)00247-2, PMID: 11695437

[ref51] Stevens R. J. Laughlin R. J. (1998). Measurement of nitrous oxide and di-nitrogen emissions from agricultural soils. Nutr. Cycl. Agroecosys. 52, 131–139. doi: 10.1023/A:1009715807023

[ref52] Takeda H. Takahashi N. Hatano R. Hashidoko Y. (2012). Active N_2_O emission from bacterial microbiota of Andisol farmland and characterization of some N_2_O emitters. J. Basic. Microb. 52, 477–486. doi: 10.1002/jobm.201100241, PMID: 22144290

[ref53] Talbot J. M. Bruns T. D. Smith D. P. Branco S. Glassman S. I. Erlandson S. (2014). Endemism and functional convergence across the NorthAmerican soil mycobiome. Proc. Natl. Acad. Sci. U. S. A. 111, 6341–6346. doi: 10.1073/pnas.1402584111, PMID: 24733885 PMC4035912

[ref54] Tian C. Zhou X. Liu Q. Peng J. W. Wang W. M. Zhang Z. H. (2016). Effects of a controlled–release fertilizer on yield, nutrient uptake, and fertilizer usage efficiency in early ripening rapeseed (*Brassica napus* L.). J. Zhejiang Univ. Sci. B 17, 775–786. doi: 10.1631/jzus.B1500216, PMID: 27704747 PMC5064171

[ref55] Wang W. Akhtar K. Ren G. Yang G. Feng Y. Yuan L. (2019). Impact of straw management on seasonal soil carbon dioxide emissions, soil water content, and temperature in a semi-arid region of China. Sci. Total Environ. 652, 471–482. doi: 10.1016/j.scitotenv.2018.10.207, PMID: 30368177

[ref56] WRB (2015). World Reference Base for Soil Resources 2014: International Soil Classification Systems for Naming Soils and Creating Legends for Soil Maps (Update 2015). In Food and Agriculture Organization of the United Nations. Available at: http://www.fao.org/soils-portal/soil-survey/soil-classification/world-reference-base/en/

[ref57] You Y. Wang J. Huang X. Tang Z. Liu S. Sun O. J. (2014). Relating microbial community structure to functioning in forest soil organic carbon transformation and turnover. Ecol. Evol. 4, 633–647. doi: 10.1002/ece3.969, PMID: 25035803 PMC4098142

[ref1001] Yu N. N. Ren B. Z. Zhao B. Liu P Zhang J. W. (2021). Leaf-nitrogen status affects grainyield formation through modification of spike differentiation in maize. Field CropsRes. 271:108238. doi: 10.1016/j.fcr.2021.108238

[ref1002] Yu N. N. Ren B. Z. Zhao B. Liu P Zhang J. W. (2022). Optimized agronomicmanagement practices narrow the yield gap of summer maize through regulatingcanopy light interception and nitrogen distribution. Europ. J. Agron. 137:126520. doi: 10.1016/j.eja.2022.126520

[ref58] Zhang K. Chen L. Li Y. Brookes P. C. Xu J. Luo Y. (2020). Interactive effects of soil pH and substrate quality on microbial utilization. Eur. J. Soil Biol. 96:103151. doi: 10.1016/j.ejsobi.2020.103151

[ref59] Zhao H. L. Tian X. H. Jiang Y. H. Zhao Y. Si B. C. (2021). Effect of combining straw-derived materials and wood ash on alkaline soil carbon content and the microbial community. Eur. J. Soil Sci. 72, 1863–1878. doi: 10.1111/ejss.13081

[ref60] Zheng L. Wu W. Wei Y. Hu K. (2015). Effects of straw return and regional factors on spatio-temporal variability of soil organic matter in a high–yielding area of northern China. Soil Till. Res. 145, 78–86. doi: 10.1016/j.still.2014.08.003

[ref61] Zhu L. Q. Hu N. J. Zhang Z. W. Xu J. L. Tao B. R. Meng Y. L. (2015). Short-term responses of soil organic carbon and carbon pool management index to different annual straw return rates in a rice-wheat cropping system. Catena 135, 283–289. doi: 10.1016/j.catena.2015.08.008

